# Microsurgical resection of brainstem cervicomedullary ganglioglioma: operative video and technique of creating a surgical pseudoplane for near-total resection

**DOI:** 10.3171/2019.10.FocusVid.19413

**Published:** 2019-10-01

**Authors:** James K. Liu, Vincent N. Dodson

**Affiliations:** Department of Neurological Surgery, Center for Cerebrovascular and Skull Base Surgery, Neurological Institute of New Jersey, Rutgers University, New Jersey Medical School, RWJ Barnabas Health, Newark and Livingston, New Jersey

**Keywords:** brainstem, cervicomedullary, ganglioglioma, pseudoplane, video

## Abstract

Cervicomedullary gangliogliomas are rare low-grade neoplasms of the brainstem. They can be challenging lesions to resect due to the eloquent location in the brainstem. In some instances, the absence of a clear surgical plane between the tumor and normal neural tissue can prohibit a complete resection. Therefore, it is important to leave a thin rim of residual tumor at the tumor-brainstem interface in order to avoid irreversible neurological injury. In this operative video, the authors demonstrate the technique to develop a surgical pseudoplane using sharp microdissection for a cervicomedullary brainstem ganglioglioma without a clear interface between the tumor and normal neural tissue. This strategy allowed for radical near-total resection of the tumor, thereby maximizing the extent of removal while preserving neurological function. Postoperatively, the patient had normal neurological function and returned to work without any disability. In summary, due to the lack of a clear surgical dissection plane, a pseudoplane near the surgical interface can be performed using sharp dissection to facilitate radical near-total resection.

The video can be found here: https://youtu.be/biD4G1Hh0yk.

## Transcript

### 0:20–0:38 Title

This is Dr. James Liu, and I’ll be demonstrating an operative video of microsurgical resection of a brainstem cervicomedullary ganglioglioma, and the technique for creating a surgical pseudoplane for near-total resection when there is no clear interface between the tumor and normal neural tissue.

### 0:38–0:58 Patient history

The patient is a 22-year-old female who was involved in a motor vehicle accident and was evaluated for posttraumatic headaches and concussion. She reported worsening headaches, numbness, and tingling of the occipital region and upper and lower extremities, especially in the dorsum of the hands. The remainder of her exam was nonfocal.

### 0:58–1:47 Preoperative imaging

T2 sagittal MRI demonstrated a dorsal hyperintense mass expanding the cervicomedullary junction extending from the medulla to C2, occluding the foramen magnum. There is a thin rim of neural tissue ventral to the mass. The mass was hyperintense on FLAIR but did not enhance on T1 postgadolinium images. Axial T2 images show the hyperintense mass dorsal to the medulla and spinal cord. Again, there is a thin rim of neural tissue (white arrows) that is compressed by the dorsal tumor. Differential diagnosis of cervicomedullary brainstem tumors includes juvenile pilocytic astrocytoma, ganglioglioma, diffuse astrocytoma, and ependymoma.

### 1:47–2:23 Patient positioning and skin incision

We performed a midline posterior craniocervical approach in the standard prone Concorde position with a midline skin incision. Intraoperative navigation and neuromonitoring are used. Because of the eloquent location of the tumor in the brainstem, we monitored facial nerve and lower cranial nerve EMGs, brainstem auditory evoked responses, and somatosensory and motor evoked potentials. After making a midline skin incision, subperiosteal elevation of the soft tissues exposes the subocciput down to C2.

### 2:23–2:39 Surgical approach and dural incision

We performed a suboccipital craniectomy, and C1 and C2 laminectomies to adequately expose the cervicomedullary junction tumor. The atlantooccipital ligament is excised, and the dura is opened sharply in a Y-shaped fashion.

### 2:40–7:32 Intradural exposure and resection of the cervicomedullary tumor

The dural leaflets are tacked up and the arachnoid over the cisterna magna is incised sharply and continued inferiorly over the cervicomedullary junction. A dorsally exophytic grayish-looking tumor is visualized just beneath both cerebellar tonsils. The expansile tumor started at the obex rostrally and extended inferiorly to C2. Starting from the rostral pole of the tumor, we performed a midline myelotomy with a bipolar cautery followed by a no. 11 blade. We initially took a biopsy of the more rostrally dorsally exophytic component, which came back as pilocytic astrocytoma with lots of Rosenthal fibers.

We then proceeded to debulk the tumor using an ultrasonic aspirator. This allowed collapse of the tumor cavity so that we could work around the superolateral aspect of the tumor. We then elevated both tonsils and lysed the arachnoid adhesions on the right side of the tumor. Here we can see cranial nerves XI and XII on the right side. There was an area that appeared to be the plane between the tumor and the brainstem, and we debulked the tumor to this margin using the ultrasonic aspirator. As we came down on the rostral pole of the tumor, we visualized the rhomboid fossa and carefully trimmed the tumor close to the neural-tumor interface while preserving the structures of the rhomboid fossa.

We then worked around the right superolateral surface of the tumor and used the ultrasonic aspirator to debulk the tumor until we were near the interface of the tumor and brainstem.

The tumor was noted to be firm and without a clear discernible plane of dissection between the tumor and normal neural tissue. Therefore, it is important to leave a thin rim of tumor tissue at the tumor-brainstem interface, so as to avoid irreversible neurological injury.

We then worked around the inferior pole of the tumor and identified where it was exiting exophytically on the dorsal aspect. We used bipolar cautery to devascularize the tumor vessels. Sharp dissection using microscissors was then performed to create a plane on the inferior pole of the tumor. We carefully peeled the normal cord away from the tumor surface using a disc dissector. However, the surgical interface between the tumor and normal cord was indistinct. So, we therefore used microscissors to create a pseudoplane to dissect the tumor away from the left lateral margin of the spinal cord. This plane was continued towards the inferior pole of the tumor.

Again, it was evident that the tumor was adherent at the interface between the tumor and normal spinal cord. Therefore, sharp dissection with microscissors using a combination of spreading and cutting was very effective in creating the plane of dissection. Again, we chose to leave a thin rim of tumor that was adherent to the tumor–spinal cord interface to avoid neurological injury. This surgical plane was carried along the ventral aspect of the tumor.

Once the tumor was separated from the ventral aspect of the spinal cord, the free component of the tumor could be aspirated.

Now that the ventral margin of the resection cavity was defined, the ultrasonic aspirator can be used to debulk the tumor down to the depths of the pseudoplane that was created.

The last component of remaining tumor was situated in the inferolateral aspect of the tumor cavity, which was readily dissected off using the ultrasonic aspirator with attention to the surgical interface.

This technique of creating a pseudoplane near the surgical interface allowed us to achieve a near-total resection without neurological injury. The fourth ventricle and obex were all preserved. There were no changes in the neuromonitoring throughout the case. Here we can see the right vertebral artery (VA) and cranial nerve XI.

### 7:32–8:03 Closure

Hemostasis was achieved and the surgical cavity was lined with Surgicel. A watertight dural closure was performed using an AlloDerm graft to repair the dural defect with running sutures. A small fat graft, harvested from the subcutaneous tissue of the neck, was placed at the inferior limb of the closure, and a cranioplasty was placed using a suboccipital plate to repair the skull defect.

### 8:03–8:56 Postoperative course

Immediate postoperative axial FIESTA imaging shows near-total resection of the tumor with minimal residual tumor lining the floor of the resection cavity. This was felt to be a near-total resection, greater than 95%. There was no recurrence or progression on the 1-year postoperative MRI.

The final pathology was ganglioglioma, WHO grade I. Although the vast majority showed pilocytic astrocytoma morphology, the presence of dysmorphic neoplastic ganglion cells is diagnostic for ganglioglioma.

Postoperatively, the patient experienced improvement in headaches, numbness, and tingling in the limbs, although it was persistent in the left hand. Motor strength, gait, and balance remained normal, and she returned to work without any disability.

### 8:56–9:24 Conclusion

In summary, cervicomedullary gangliogliomas can be surgically challenging lesions to resect due to the eloquent location and possible absence of a clear surgical plane (Lang et al., 1993; Weiner et al., 1997; Kim et al., 2014; McAbee et al., 2015; Puget et al., 2015; Janjua et al., 2017). The technique of creating a pseudoplane near the surgical interface using sharp dissection facilitates radical near-total resection to maximize extent of resection while preserving neurological function.

## Supplemental Information

Supplementary Figs. 1 and 2
