# Supplementary material for: Microsurgical resection of brainstem cervicomedullary ganglioglioma: operative video and technique of creating a surgical pseudoplane for near-total resection
Source: Neurosurg Focus Video. 2019 Oct 1;1(2):V13. doi: 10.3171/2019.10.FocusVid.19413 (PMC9557339; doi:10.3171/2019.10.FocusVid.19413)
Supplement: Supplementary Figs. 1 and 2 [file SupplementaryFigs1and2_OCtFocusVid19413.pdf]

Supplemental material

Microsurgical resection of brainstem cervicomedullary ganglioglioma: operative video and technique of creating a surgical pseudoplane for near-total resection

James K. Liu, MD and Vincent N. Dodson, BS

http://thejns.org/doi/abs/10.3171/2019.10.FocusVid.19413

DISCLAIMER *Neurosurgical Focus: Video* acknowledges that the following section is published verbatim as submitted by the authors and did not go through either the journal's peer-review or editing process.

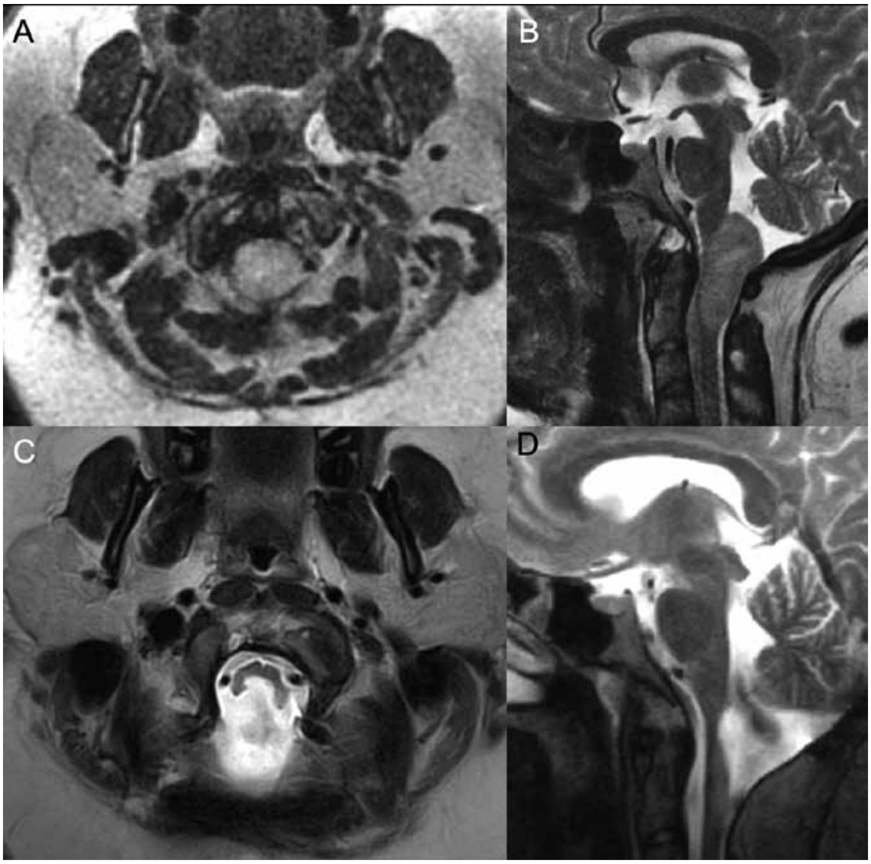

FIGURE 1. Preoperative axial (A) and sagittal (B) T2 MRI demonstrate a hyperintense mass compressing the brainstem at the cervicomedullary junction. One-year postoperative axial (C) and sagittal (D) T2 MRI demonstrate near-total resection (> 95%) of the tumor with minimal residual tumor lining the floor of the resection cavity.

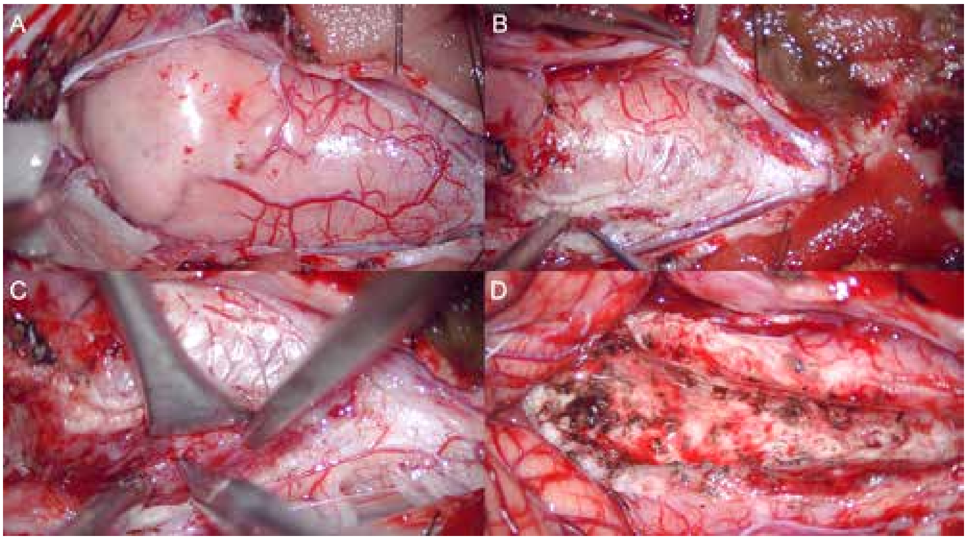

FIGURE 2. A: Intraoperative image demonstrating the ganglioglioma, which appears as a grayish-looking tumor inferior to the cerebellar tonsils. B and C: The interface between tumor and normal neural tissue is not clear, so a pseudoplane is created using sharp dissection to achieve radical near-total resection. D: Only a thin rim of tumor remained on the resection bed after near-total resection.
